# Postpartum deep vein thrombosis resolved by catheter-directed thrombolysis: A case report

**DOI:** 10.1097/MD.0000000000016052

**Published:** 2019-06-14

**Authors:** Po-Wei Chen, Ping-Yen Liu

**Affiliations:** a Division of Cardiology, Department of Internal Medicine, National Cheng Kung University Hospital; b Institute of Clinical Medicine, College of Medicine, National Cheng Kung University, Tainan, Taiwan.

**Keywords:** catheter-directed thrombolysis, postpartum, thrombosis, venous thrombosis

## Abstract

**Rationale:**

Postpartum deep vein thrombosis is a unique condition in diagnosis and treatment. Rivaroxaban, a novel oral anticoagulant, is indicated for acute deep vein thrombosis, but limited data have been reported for postpartum women. Catheter-directed thrombolysis is a common procedure for treating acute deep vein thrombosis, but it is rarely used for postpartum patients, especially after more than 3 months.

**Patient concerns:**

A 31-year-old Asian woman suffered from progressive erythematous swelling and local heat of the left lower limb after twin delivery.

**Diagnoses:**

Venous duplex ultrasound examination showed thrombus formation in the left femoral vein and popliteal vein with reduced compressibility. After standard treatment of novel oral anticoagulant therapy for 4 months, we observed only partial improvement of the symptoms, and the condition deteriorated after her ordinary activities.

**Interventions:**

Venography was performed and a large amount of thrombus lining from left femoral vein to left iliac vein was noted with total occluded left common iliac vein. After catheter-directed thrombolysis and balloon dilatation, better flow was regained and her symptoms improved completely after procedure.

**Outcomes:**

During a 1-year follow-up without medication, the patient did not complain about leg swelling, exercise aggravation, or any other post-thrombotic symptoms.

**Lessons:**

Pregnancy seems to be a transient provoking factor for deep vein thrombosis, but it is sometimes refractory even during the postpartum period.

Follow-up imaging studies should be encouraged to confirm the vessel condition, particularly for applying down-titration or discontinuation strategies of medication.

Catheter-directed thrombolysis could be considered as an alternative method for postpartum iliofemoral deep vein thrombosis. Postpartum women usually have favorable functional status and lower bleeding risk.

Rivaroxaban is a favorable choice for deep vein thrombosis, but its use in postpartum women is still controversial, and evidence of its effectiveness is not available. Thus, endovascular intervention can be a relatively safe therapy, in addition to anticoagulation therapy for premenopausal patients with recurrent deep vein thrombosis.

## 1 Introduction

Venous thromboembolism (VTE), comprising deep vein thrombosis (DVT) and pulmonary embolism, is a common disorder with an annual incidence of approximately 1 case per 1000 persons.^[1]^ Nearly two-thirds of VTE episodes manifest as DVT and one-third as pulmonary embolism with or without DVT. The background risk factors for VTE must be explored to determine whether it is modifiable. Pregnancy and the puerperium are two of the most well-established provoked factors for VTE.^[2]^ However, the treatment strategy for such patients remains uncertain.

During the third trimester and during the first 2 weeks following delivery, women have a relatively high risk of VTE, which is the leading cause of maternal death in Western countries.^[3]^ The subjective clinical assessment of DVT is usually difficult during pregnancy, with a limited number of women having the opportunity to complete the diagnosis confirmed using objective exams. In general, women have bilateral leg edema developed at the third trimester, even lasting to the postpartum period, thus masking the signs of DVT over the legs. In addition, bed rest and hormonal therapy during pregnancy are often prescribed for symptomatic relief, and these management methods may increase the risk of DVT. With multiple reversible risk factors for DVT in such patients, the physiological response of the pregnancy process remains the most inevitable and easily ignored factor for hypercoagulopathy.^[3]^

Current guidelines recommend the use of subcutaneous low-molecular-weight heparin, rather than intravenous unfractionated heparin or vitamin K antagonists, for women during pregnancy.^[3]^ Notably, anti-Xa inhibitors, which are among the novel oral anticoagulant (NOAC), are not currently recommended for pregnant women.^[2,3]^ Their use in postpartum women is also controversial, and evidence of their effectiveness in such women is not available. In this paper, we report a case of a postpartum woman diagnosed as having DVT with possible NOAC treatment failure, who was rescued using catheter-directed thrombolysis.

## 2 Patient information and clinical findings

A 31-year-old Asian woman was found to have twin pregnancy during her first gravida. She initially presented frequent vaginal bleeding, and her obstetrician prescribed oral progesterone at a dosage of 300 to 400 mg per day (Utrogestan) and intramuscular progesterone at a dosage of 125 mg/amp per week (Progeston Depot-s) since her 8th week of gestation. However, because of early uterine contraction and cervical incontinence, she started to take additional ritodrine at 10 mg 6 times daily (Yutopar) and rectal indomethacin since her 18th week of gestation. After Mcdonald cerclage was performed at the 21st week of gestation, she restricted her daily activities at home and rested in bed most of the time, following her obstetrician's advice. A cesarean section was then arranged at the 32nd week due to frequent uterine contractions. However, progressive erythematous swelling and local heat of the left lower limb were found on the 14th day after delivery. In addition, she denied dyspnea, chest pain, fever, chills, cough, and hemoptysis.

Venous duplex ultrasound examination showed thrombus formation in the left femoral vein and popliteal vein with reduced compressibility. Under the impression of DVT with VTE, a regimen of rivaroxaban (15 mg) twice daily was prescribed after discussion by mode of shared decision making with families and patient herself.

After oral anticoagulation therapy with additional medical compression stocking, her leg swelling was ameliorated gradually 3 days later. On the basis of current clinical trial and treatment guidelines, we switched the regimen to rivaroxaban (20 mg) once daily after 21 days.

After 3 months of anticoagulation therapy, we observed only partial improvement of the symptoms, and the condition deteriorated after her ordinary activities. Repeated venous duplex ultrasound examination revealed residual thrombus in the left femoral vein. For further evaluation and management, we performed invasive venous angiography.

## 3 Interventions

**Figure 1:**
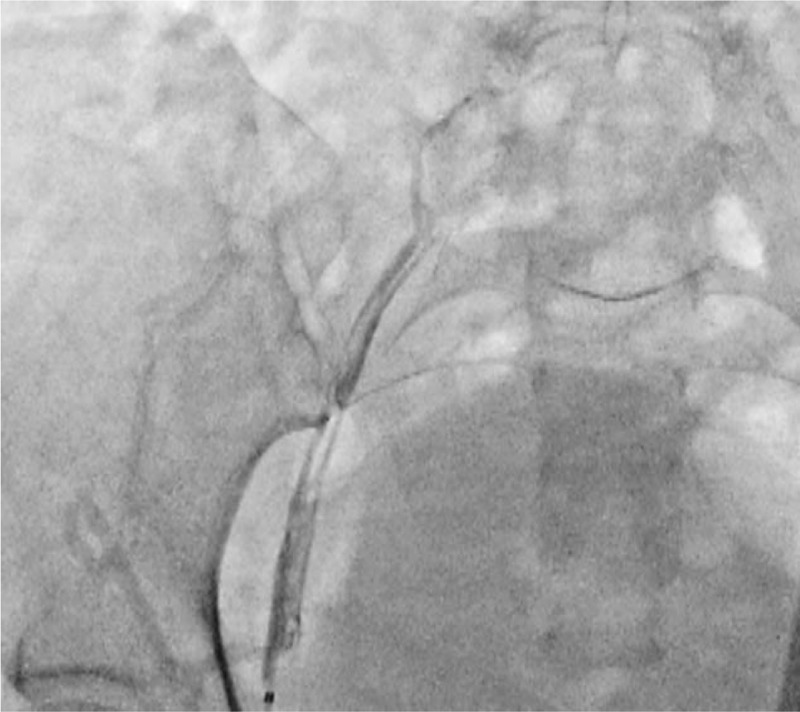
Procedure day 1. Venogram obtained from the left popliteal vein showed thrombus formation in the left iliofemoral vein and total occlusion in the left common iliac vein (Fig. 1).

Venography was performed through the left popliteal vein after popliteal vein cannulation under ultrasound guidance. We observed a large amount of thrombus lining from the femoral vein to the iliac vein. We also found total occlusion along the external iliac vein to the inferior vena cava (IVC) with abundant collateral vessels (Fig. 1). The infusion catheter was then inserted for catheter-directed thrombolysis with urokinase (60000 IU/h), concomitant with systemic heparinization.

**Figure 2:**
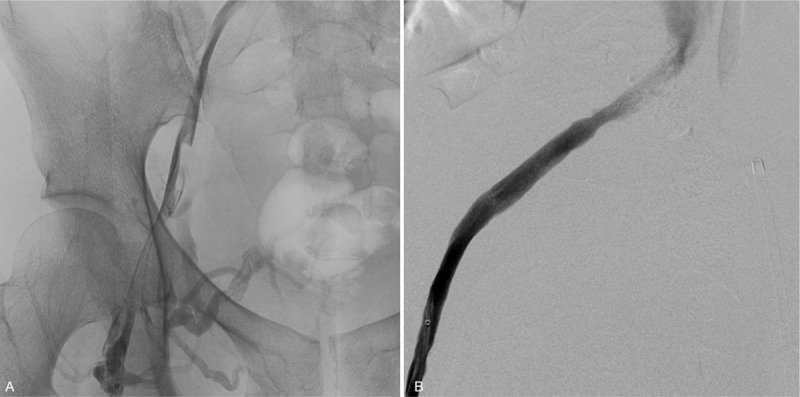
Procedure day 2. After catheter-directed thrombolysis and balloon dilatation, residual thrombus was still noted with collateral veins (Fig. 2A). Residual stenosis was noted in the proximal part of the external iliac vein (Fig. 2B).

Twenty-four hours following venography, we observed residual thrombosis with poor antegrade flow in the main trunk. Subsequently, percutaneous transluminal angioplasty was performed from the common femoral vein to the common iliac vein after IVC filter placement. An infusion catheter for catheter-directed thrombolysis with the same dosage for 1 more day was arranged because of the residual thrombosis (Fig. 2).

**Figure 3:**
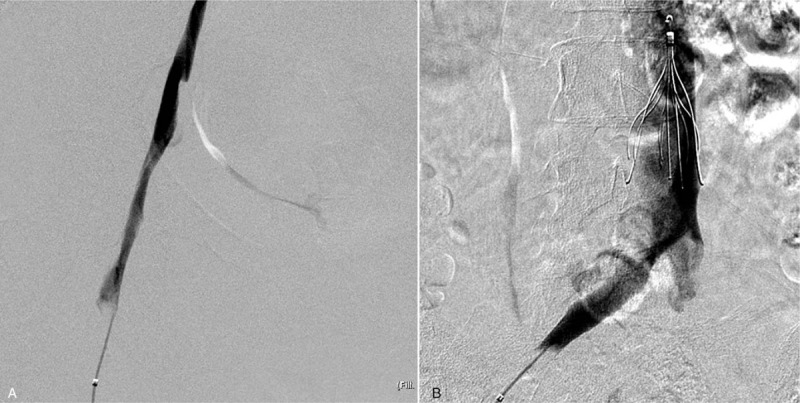
Procedure day 3. After 1 more day of catheter-directed thrombolysis and larger balloon dilatation, improved main-trunk flow of femoral vein was noted (Fig. 3A). Residual stenosis in the proximal part of the external iliac vein improved with more favorable flow (Fig. 3B).

After 2 days of thrombolysis and balloon dilatation, final venography revealed improved antegrade main-trunk flow with less than 30% residual luminal area narrowing (Fig. 3). The patient felt free of symptoms completely after endovascular intervention.

## 4 Follow-up and outcomes

**Figure 4:**
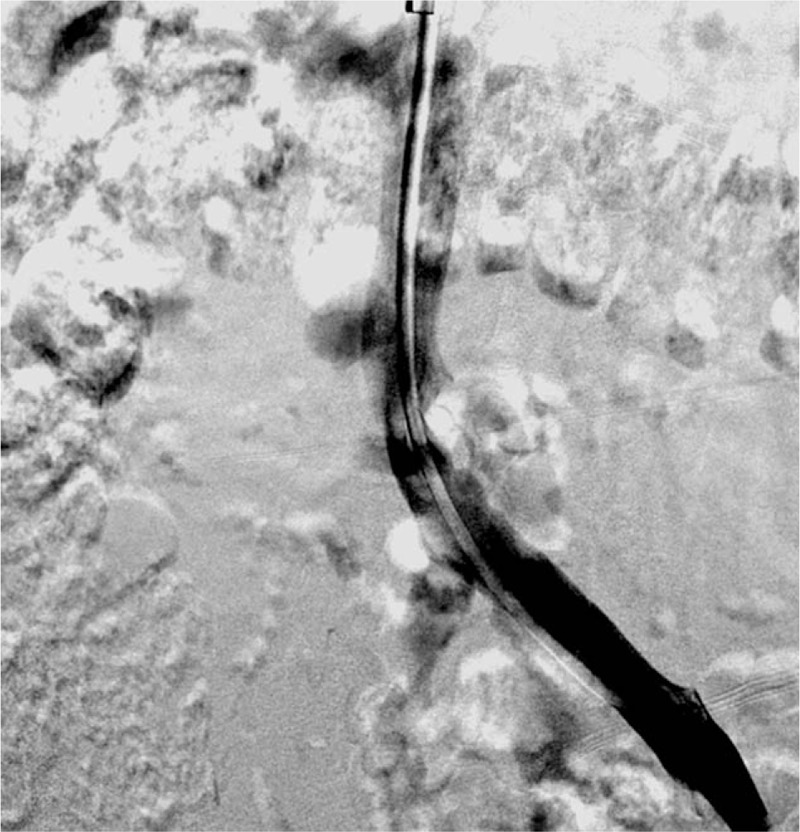
After 2 weeks, follow-up venography was performed before IVC filter retrieval.

One month after the intervention, IVC filter retrieval was arranged, and patent iliac-femoral venous flow was confirmed by a subsequent venography procedure (Fig. 4). Six months later, a patent femoral vein without remarkable thrombus was noted through venous duplex ultrasound examination, and oral anticoagulation therapy was discontinued. During a 1-year follow-up without medication, the patient did not complain about leg swelling, exercise aggravation, or any other post-thrombotic symptoms.

## 5 Discussion

Catheter-directed thrombolysis is a common procedure for acute DVT, but it is rarely used for postpartum patients, especially after more than 3 months. Herein, we report a case of a postpartum woman initially diagnosed as having DVT with possible NOAC treatment failure, who was eventually rescued using catheter-directed thrombolysis 5 months later.

Based on the guideline for VTE in Taiwan, pregnancy-related VTE is a specific issue; nevertheless, no recommendations are available for postpartum VTE.^[4]^ The European Society of Cardiology guideline in 2014 recommend that anticoagulant treatment for pregnancy-related VTE should be administered for at least 6 weeks after delivery and with a minimum overall treatment duration of 3 months.^[2]^ Furthermore, warfarin can be administered to breast-feeding women, but NOAC is not mentioned for the postpartum period in the current guidelines.^[2,4]^

The EINSTEIN-DVT trial, a phase III randomized controlled study, established an approved indication about rivaroxaban for the treatment of acute DVT.^[5]^ However, pregnant patients have almost always been excluded in clinical trials, including the EISTEIN-DVT study, although the EISTEIN-DVT study protocol did not clearly restrict postpartum patients. The study enrolled 6 postpartum patients, representing 0.3% of the rivaroxaban treatment group, but no subgroup analysis was available.^[5]^ Current guidelines recommend 3 to 6 months of anticoagulant therapy for women with provoked factors for VTE.^[2,4]^ However, no suggestion is available for those with NOAC treatment failure. Furthermore, catheter-directed thrombolysis is reserved for patients with massive iliac-femoral DVT or anticoagulation therapy failure.^[6]^

Our patient was diagnosed as having DVT 14 days immediately after delivery. Her possible provoked factors included hormonal drugs, immobilization, and pregnancy, especially twin pregnancy. After smooth delivery, these provoked factors were completely corrected. On the basis of current guidelines, 3 months of anticoagulation therapy should be recommended.^[2]^ Moreover, the patient's symptoms improved just after rivaroxaban use; no more invasive procedure was considered initially. However, her symptoms recurred as leg swelling, which was aggravated after walking or standing and relieved after resting or raising the leg. After the failure of compression stocking, catheter-directed thrombolysis was considered as an alternative method for treating chronic DVT, and this procedure was safe for the patient who had favorable functional status and low bleeding risk. Eventually, follow-up venography showed not only one blood clot above the knee but also a total occluded lesion in the common iliac vein.

No randomized controlled trials have tested the comparative effectiveness and safety of pharmacomechanical thrombectomy in the management of patients with DVT.^[6]^ In addition, in previous case series, catheter-directed thrombolysis was safe and effective for acute iliofemoral DVT in the postpartum period.^[7]^ However, there are only a few reported pregnancy-provoked cases of patients who received endovascular intervention because of anticoagulation therapy failure after 3 to 6 months.^[7–9]^

This case provides several clinical implications. First, pregnancy seems to be a transient provoking factor, but it is sometimes refractory even during the postpartum period. Second, follow-up imaging studies should be encouraged to confirm the vessel condition, particularly for applying down-titration or discontinuation strategies of medication. Third, catheter-directed thrombolysis could be considered as an alternative method for treating postpartum iliofemoral DVT. Postpartum women usually have favorable functional status and lower bleeding risk. Rivaroxaban is a favorable choice for DVT, but its use in postpartum women is still controversial, and evidence of its effectiveness is not available. Thus, endovascular intervention can be a relatively safe therapy, in addition to anticoagulation therapy for premenopausal patients with recurrent DVT.

## Author contributions

**Conceptualization:** Po-Wei Chen, Ping-Yen Liu.

**Data curation:** Po-Wei Chen.

**Project administration:** Po-Wei Chen.

**Resources:** Po-Wei Chen.

**Supervision:** Ping-Yen Liu.

**Writing – original draft:** Po-Wei Chen.

**Writing – review & editing:** Po-Wei Chen.

Po-Wei Chen orcid: 0000-0003-2300-0698.
